# Editorial: Pharmacology of autoimmune and neuroinflammatory disease from a preclinical and clinical perspective

**DOI:** 10.3389/fphar.2023.1228406

**Published:** 2023-06-13

**Authors:** Martin Šíma, Andrea Laslop, John Joseph Borg, Silvester Poništ, Daniela Melchiorri, František Dráfi

**Affiliations:** ^1^ Institute of Pharmacology, First Faculty of Medicine, Charles University in Prague and General University Hospital in Prague, Prague, Czechia; ^2^ Agency for Health and Food Safety (AGES), Vienna, Austria; ^3^ Malta Medicines Authority, Sir Temi Żammit Buildings, Malta Life Sciences Park, San Gwann, Malta; ^4^ School of Pharmacy, Department of Biology, University of Tor Vergata, Rome, Italy; ^5^ Institute of Experimental Pharmacology and Toxicology, Centre of Experimental Medicine SAS Bratislava, Bratislava, Slovakia; ^6^ Department of Physiology and Pharmacology, Sapienza University, Rome, Italy; ^7^ State Institute for Drug Control, Bratislava, Slovakia

**Keywords:** Alzheimer's disease, neuroinflammation, RIPK2, Behcet’s disease, oligodeoxynucleotides, Sjögren’s syndrome, immunotargets, immunopharmacology

## 1 Introduction

Current insights into the immunopharmacology of autoimmune diseases and neuroinflammation treatment are described in this Research Topic from a pre-clinical and clinical point of view with the aim to contribute to the development of more effective and safe treatments.

## 2 Research topic articles

Receptor-interacting serine/threonine kinase 2 (RIPK2) was first reported in 1998 in association with NF-κB activation and cell death (McCarthy et al., 1998). As a dual-specific kinase downstream of the nucleotide-binding oligomerization domain 1 and 2 signaling pathways (a kinase that is also involved in CD4^+^ T-cell proliferation and T-helper cell development), RIPK2 plays an important role in innate and adaptive immunity. Recent findings have proved that RIPK2 activation is a key factor in the pathophysiology of various autoimmune diseases such as inflammatory bowel diseases (IBDs), multiple sclerosis, rheumatoid arthritis, or Behcet’s disease (BD). The possibility of modulating RIPK2 function in treating autoimmune diseases has thus become a focus of research interest, although the broad tissue expression of RIPK2 may negatively impact the safety and tolerability profile of drugs targeting this serine/threonine kinase. The review of Pham et al. summarized recent progresses and Research Topic in the development of new RIPK2 inhibitors and degraders, discussing several promising drug candidates that are now in pre-clinical or clinical development mainly for the treatment of IBD.

Among autoimmune diseases, BD is a rare disease that causes inflammation of blood vessels throughout the body and may result in mouth sores, eye inflammation, skin rashes, and lesions or genital ulcers. Although the therapeutic armamentarium for BD has considerably grown in the past decade, the available options are still suboptimal, and improvements in our knowledge of disease mechanisms are needed. In particular skin lesions significantly contribute to the burden of the disease, but research on BD analyzing skin tissue was not performed till today. Liu et al. by combining RNA-sequencing and single-cell sequencing data analyses, have started to shed light on the predominant infiltrating monocyte subtypes, differentially expressed genes and pathways observed in the BD patients’ lesioned skin. Further investigations on the contributions of these cells, genes, and pathways to disease development will help understanding and hopefully better targeting the skin manifestations of BD.

Other tools that have attracted attention in recent years for their potential to treat autoimmune diseases are inhibitory oligodeoxynucleotides (ODNs). These short synthetic single-stranded DNA molecules can fold into complex structures, which may bind to several targets, allowing a better control of the immune system activation (Hammond et al., 2021). Some ODN-containing drugs have already been approved for marketing and are probably among the most promising tools for genetically targeted therapy. However, the development of inhibitory ODNs still faces problems, such as easy degradation by ribozymes in the blood or rapid renal clearance due to low molecular weight, and off-target effects. The review of Wang et al. compiles the current evidence on the possibilities of chemical modification, various mechanisms of action, and the potential for the treatment of autoimmune diseases.

Primary Sjögren’s syndrome (pSS) is a systemic and chronic autoimmune disease leading to exocrine gland (mainly salivary and lacrimal glands) dysfunction and various extra-glandular manifestations. Serological and histopathological assessments show increased serum autoantibodies and lymphocytic infiltration in exocrine glandular tissues, respectively (Bowman, 2018). However, the existence of a causal relationship between lymphocytic infiltration and salivary gland dysfunction is still controversial. In their meta-analysis, Wang et al. found no statistically significant effect of several biologics, targeting different aspects of inflammation, in ameliorating saliva production in the general pSS population. A statistically significant difference was, however, found in the number of SAE between biologics and control groups, independently of treatment duration or type of system disorder. Several factors may affect the safety of biologics including the type of biologics, the dose, the administration route, and administration frequency. The observed generalized worsening in pSS patients’ conditions after treatment calls for a more cautious design of clinical trials with biologics.

Despite extensive research, no disease-modifying therapeutic option able to prevent, cure, or halt the progression of Alzheimer’s disease (AD) is currently available. Although the monoclonal antibody lecanemab, targeting amyloid β (Aβ), was recently approved by both FDA and EMA, and a second anti-Aβ antibody, donanemab, is currently in phase 3 development, the magnitude of their clinical effect is limited, suggesting that additional pathological mechanisms may contribute to the disease. In their review, Melchiorri et al. propose that neuroinflammation might play a key role in AD’s pathogenesis, synergistically with Aβ and neurofibrillary tangle cascades. They thus discuss the possibility of a multi-target therapy of AD combining anti-amyloid drugs with agents targeting neuroinflammation. The authors described 18 investigational drugs, targeting neuroinflammation, currently under clinical investigation in AD, as well as 20 patents retrieved from the WIPO-IP portal. Several promising candidates emerge from the authors’ search, including treatments targeting CNS-resident microglia and agents modulating kinase signaling. However, the optimization of combination therapy with anti-inflammatory drugs could be complex. The efficacy of anti-inflammatory therapies could be restricted to a certain time frame in the disease course, which may not be easy to intercept in each patient. The design of clinical trials evaluating combination therapy is also complex, and there is the additional need for cooperation between pharmaceutical industries. A possible way through is suggested to be consortia between industries and academia, which use uniform protocols and outcome measures, and allowing treatment arms to be added or dropped based on interim analyses of outcomes.

## 3 Conclusion

In autoimmune diseases and neuroinflammatory diseases, the condition arises through aberrant human adaptive or innate immune systems reactions. The biological mechanisms underlying activation and regulation of immune system and inflammation are complex, time dependent, and often overlapping each other; in addition, critical immune cells like monocytes and microglia may progress or cycle through different activation states, having beneficial or detrimental impacts on the disease. An example of this is AD, in which innate immunity plays different roles across different states of the disease. Moreover, some immune-mediated diseases, like BD, share the characteristics of both autoimmune diseases and autoinflammatory syndromes and have variable clinical manifestations in patients, further complicating treatment optimization. In IBDs, dysregulated immune responses couple with mucosal barrier dysfunction, disturbances in the gastrointestinal microbiota, genetic predisposition, and environmental factors, resulting in a considerable fraction of patients who do not respond to available treatments or lose response, which calls for new therapeutic strategies. By introducing and discussing novel mechanisms of action and drug candidates for the treatment of autoimmune and neuroinflammatory diseases, the different contributions to this Research Topic provide an overview of potential therapeutic strategies for a clinically diverse group of conditions for which there are no current cures.
